# A Detailed Characterisation of Appetite, Sensory Perceptional, and Eating-Behavioural Effects of COVID-19: Self-Reports from the Acute and Post-Acute Phase of Disease

**DOI:** 10.3390/foods10040892

**Published:** 2021-04-19

**Authors:** Nora Chaaban, Alexander Teymour Zadeh Baboli Høier, Barbara Vad Andersen

**Affiliations:** Food Quality Perception and Society, Department of Food Science, Aarhus University, Agro Food Park 48, DK-8200 Aarhus N, Denmark; ncho_on@hotmail.com (N.C.); alexanderhoier@gmail.com (A.T.Z.B.H.)

**Keywords:** COVID-19, appetite, sensory perception, eating behaviour, self-reports

## Abstract

Sensory perception alterations are common in relation to COVID-19 disease, but less is known about the characteristic of the sensory alterations, and how they associate with alterations in appetite and eating behaviour. The current study aims to investigate the acute and long-term effects of COVID-19 disease on (1) the desire for food, hunger, and satiety sensations; (2) smell, taste, and flavour perception; (3) meals and intake of food types; and (4) the frequency of commonly applied strategies to tackle potential changes in appetite and sensory perception. An online survey was conducted among Danish adults (*n* = 102) who had experienced changes in appetite, sensory perception, and/or food-related pleasure due to COVID-19 disease. Key results include appetite-altering effects at all times during the day when suffering from COVID-19 and often associated with impaired sensory function. Severe sensory perception alterations were found, namely, for the perception of taste, ageusia > hypogeusia > hypergeusia, and for the perception of smell, anosmia > parosmia > hyposmia > hyperosmia. Eating behavioural changes included alteration in quantitative and qualitative aspects of intake. The effects were, in general, more pronounced during the acute phase of disease than during the post-acute phase. The findings illustrate the complexity by which COVID-19 affects human appetite, sensory perception, and eating behaviour, but also point to strategies to cope with these changes.

## 1. Introduction

The most common symptoms of COVID-19 disease are fever, cough, and fatigue [1,2]. Less common symptoms include sore throat and loss of smell (anosmia) and taste (ageusia), amongst other symptoms. Initially, anosmia and ageusia were not considered symptoms of COVID-19 disease. However, since the beginning of the COVID-19 pandemic, the number of patients reporting either a total or partial loss of smell and taste in connection with a SARS-CoV-2 infection has increased. Sensory dysfunction has been documented in studies utilizing (i) self-report questionnaires, namely, the National Health and Nutrition Examination Survey [3,4], the Sino-Nasal Outcome Test [5], the short-version Questionnaire of Olfactory Disorders [3,4], and questionnaires designed for the specific study in focus, e.g., [6,7,8], and (ii) objective testing, namely, taste-recognition test [8,9], smell-identification test [10,11,12] and smell-threshold test [9]. Now (April 2020), anosmia and ageusia are on the Danish health authorities’ list of common long-term effects of COVID-19 affecting people of all age groups and health, and regardless of the severity of COVID-19 illness [13]. It is estimated that around 10% of those who contract COVID-19 experience long-term symptoms (lasting for more than four weeks = ongoing symptomatic COVID-19). The proportion of those who continue to show symptoms after 12 weeks (post-COVID-19 syndrome) is still not known. Since COVID-19 is still a relatively new disease, knowledge of ongoing symptomatic COVID-19 and the post-COVID-19 syndrome is needed. The majority is expected to recover on their own, although some more slowly than others and without knowing if the symptoms will be chronic for some [13].

Although the loss of smell and taste are not critical to our health per se, perception of sensory properties is closely related to well-being since sensory perception is greatly linked to eating enjoyment, e.g., [14,15], memory recall [16], mood [17,18,19], etc. Therefore, loss of taste and smell can have a profound impact on people’s quality of life. Further, COVID-19 has been reported to be accompanied by a loss of appetite via a decreased motivation to initiate eating (desire for food) and continue eating (general hunger and enjoyment) [20]. A healthy appetite is, in general, important when recovering from diseases since nutritional choices can affect the body’s ability to prevent, fight, and recover from infections [21]. Further, good nutrition can help regain strength and reduce the likelihood of developing other health-related problems. If a lack of appetite lasts for more than a couple of days, it can cause weight loss and, in the longer term, malnutrition.

Changes in appetite due to COVID-19 have been shown to be long lasting [20], posing a risk on the quality of life and increasing the risk of malnutrition, which can negatively affect recovery. Current studies of the appetite- and sensory-related effect of COVID-19 have mainly focused on studying self-reported olfaction perception [3,5,6,8] and the biological mechanisms driving olfaction dysfunction [22,23,24]. A detailed characterisation of the appetite- and eating-behavioural changes caused by COVID-19 will broaden our understanding of the severity of the disease from a patient perspective and can help health professionals to qualify recommendations for proper nutrition during treatment and recovery of patients.

The overall objective of this study was to investigate the effect of COVID-19 disease on self-reported appetite, sensory perception, and eating behaviour in a Danish population. Specifically, the acute and long-term effects of COVID-19 on (1) the desire for food, hunger, and satiety sensations; (2) perception of smell, taste, and flavour; and (3) meals and intake of food types. A final aim was to study the frequency of commonly applied strategies to tackle potential changes in appetite and sensory perception.

## 2. Materials and Methods

### 2.1. Participants and Recruitment

Prior to data collection, the Central Denmark Region Committees on Health Research Ethics approved the study being conducted. Data were collected via an online survey running over a period of one month, November 2020. Participants (*n* = 112) were recruited from several Danish COVID-19 groups on Facebook (one group for COVID-19 patients suffering from long-term effects, and two general COVID-19 information groups) and through posts on LinkedIn and Twitter. Inclusion criteria for the study were Danish-speaking adults (above 18 years old) who had experienced changes in appetite, sensory perception, and/or food-related pleasure due to COVID-19 disease. The latter criterion was chosen to facilitate a detailed characterisation of appetite, sensory perceptional, and eating-behavioural effects of COVID-19.

Participants completing the survey were at different stages of recovery from COVID-19 disease: (i) in the acute phase being currently ill, (ii) in the post-acute phase yet still suffering from long-term effects, and (iii) in the post-acute phase and fully recovered from COVID-19 disease. This paper focuses on the results from participants in the second group, i.e., those who were in the post-acute phase yet still suffering from long-term effects of COVID-19 disease. In total, 102 participants were included in the data analysis. Table 1 shows the characteristics of the included participants. The majority of the participants were diagnosed with SARS-CoV-2 infection through a throat swab (71%). Other diagnosis methods were antibody test (11%), doctor’s assessment (10%), own assessment (4%), and other (5%). A study by Parma et al., focusing on the effect of COVID-19 on sensory impairments, found no difference in results when comparing the type of diagnosis, lab tests, and clinical assessments [8]. Participants mostly reported to experience the acute phase of COVID-19 to last between 1–4 weeks; less than 1 week (17%), between 1–2 weeks (33%), between 2–4 weeks (33%), 4–6 weeks (9%), 6–8 weeks (3%), more than 8 weeks (1%), and not stated (4%). In addition to changes in appetite, sensory perception, and eating behaviour, participants were also asked to report other symptoms experienced during the acute phase by evaluating a list of the most common symptoms of COVID-19 disease collected from a systematic review and meta-analysis study [2]. Among these symptoms, participants in the present study reported the following symptoms as the most common (only 5% and above are mentioned here): dizziness (5%), blurry vision (5%), headache (7%), difficulty in concentrating (8%), loss of taste (10%), fever (11%), and loss of smell (11%). The duration of the experienced long-term effects from COVID-19 was not explored in the present study.

### 2.2. Online Survey

The online survey focused on self-reported changes in appetite, sensory perception, and eating behaviour, as experienced in the acute and post-acute phase due to COVID-19 disease. In this study, the post-acute phase was defined as the phase in which the COVID-19 patients had not fully recovered and thereby were suffering from long-term effects, i.e., changes in appetite, sensory perception, and/or food intake. Table 2 provides a list of response variables included in the questionnaire. Throughout the survey, the participants were asked to compare their experiences during both the acute and post-acute phase with how they felt before COVID-19. By conducting this method, the effect of COVID-19 on appetite, eating behaviour, and sensory perception was explored from a subjective perspective. Appetite- and satiety-related response variables were selected from a list of mental and physical sensations developed by Murray and Vickers [25], response variables covering sensory perception were chosen to provide an overview of potential taste, orthonasal, retronasal, off-flavour, and chemesthesis perceptional alterations, eating behavioural related response variables were selected based on subjective reports of COVID-19 patients from a recent qualitative study [20], and finally, response variables regarding strategies to cope with potential changes in appetite were selected based on results from a qualitative study on the topic [20]. The response variables were evaluated by using a three- or five-point categorical scale, with the ends indicating opposite extremes depending on the type of question. The number of categories, e.g., regarding the level of detail in intensity ratings of a sensation, rely on recommendations by previous studies and an evaluation of the appropriate reflection level required of the participants. To guide this decision, participants’ replies in a qualitative study were reviewed [20]. Additionally, a “do not know/not relevant” option was included. For questions with multiple possible answers, e.g., symptoms of COVID-19 during the acute phase, a “check all that apply” format was used. Furthermore, for more specific and subjective questions, such as personal experiences with certain foods, an open-reply field was used. These open-reply fields allowed the participant to elaborate on replies and allowed a check of reliability. In the case the information provided in the open-reply field indicated that the participant has misunderstood the question, the data were removed from the analysis.

### 2.3. Data Analysis

Mean and standard error of the mean (SEM) were calculated for each question, and the number of answers in each answer option was counted. Data were illustrated in either bar charts based on the mean or as a stacked bar chart showing the distribution of answers. Student’s T-test was used to analyse significant differences between the acute and post-acute phases. For questions with three answer options, e.g., hunger and satiety sensations and food intake, Fisher’s exact test was used to determine significant differences in frequency of use of answer options between the acute phase and the post-acute phase. Lastly, descriptive statistics were used to summarise the characteristics of the participants, which are shown in Table 1, and the weight and height were used to calculate BMI. For all analyses, XLSTAT (version 2020.5.1, Addinsoft SARL, Paris, France) was used with a significant level of α = 0.05.

## 3. Results

### 3.1. Appetite

#### 3.1.1. Desire for Food

**Figure 1:**
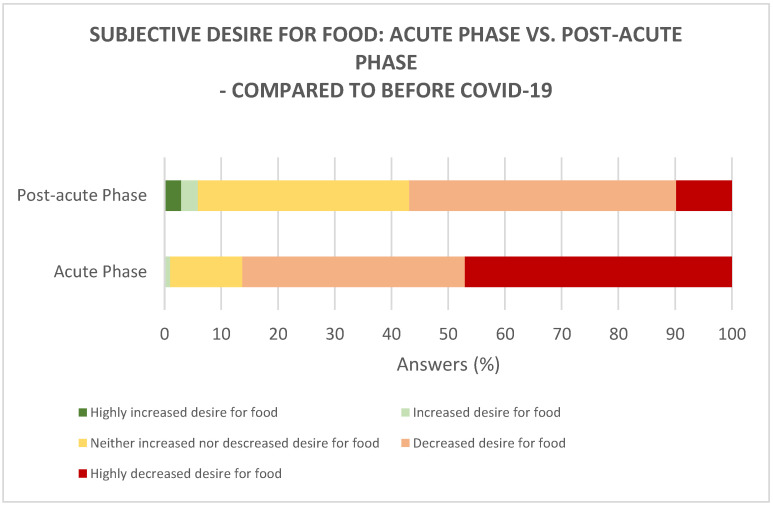
Subjective desire for food ratings in the acute phase (*n* = 102) and in the post-acute phase (*n* = 102), compared to before COVID-19.

In the acute phase, the majority of the participants (86%) experienced a decreased to a highly decreased desire for food, compared to before COVID-19 (Figure 1). In the post-acute phase, it was found that around 57% of the participants still experienced a decreased to a highly decreased desire for food; however, some participants (37%) experienced that their desire for food returned to as before COVID-19 (Figure 1). Further, in the post-acute phase, a small percentage of the participants (6%) experienced an increased to highly increased desire for food. Generally, the desire for food was significantly higher (*p* < 0.0001) in the post-acute phase, compared to the acute phase.

Among the symptoms reported to cause changes in the *desire for food* in the two phases (acute phase/post-acute phase), the most common were loss of smell (16%/17.2%), loss of taste (16%/18.2%), food not being pleasurable (11.7%/10.6%), food tasting bad or different (9.5%/14.1%), lack of hunger sensations (11.2%/10.1%), fatigue (9.5%/8.1%) and nausea (9%/7.1%). Note that only causes above 5% are mentioned.

#### 3.1.2. Time of Day

**Figure 2:**
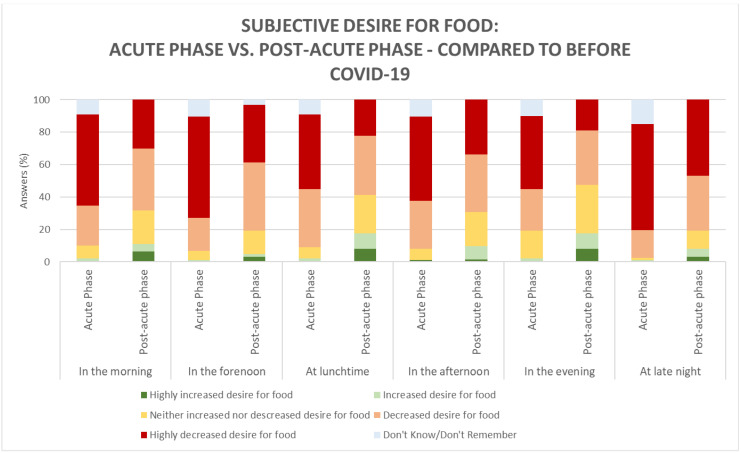
Subjective desire for food according to time of day during the acute phase (*n* = 89) and post-acute phase (*n* = 63) compared to before COVID-19.

Overall, during the acute and post-acute phases, the majority (ranging from 70.7% to 82.7% in the acute phase and 52–80.5% in the post-acute phase) of participants experienced a decreased to a highly decreased desire for food at all times during the day, compared to before COVID-19 (Figure 2). Focusing on the meals for which participants reported the highest appetite, desire for food, was generally reported to be highest around the three main meals—in the morning, at lunchtime, and in the evening—but only during the post-acute phase. The desire for food was found to be significantly higher at any time of the day in the post-acute phase, compared to the acute phase; in the morning (*p <* 0.001), in the forenoon (*p <* 0.001), at lunchtime *p <* 0.001), in the afternoon (*p <* 0.001), in the evening (*p <* 0.001), and at late night (*p <* 0.001).

#### 3.1.3. Hunger and Satiety Sensations

**Figure 3:**
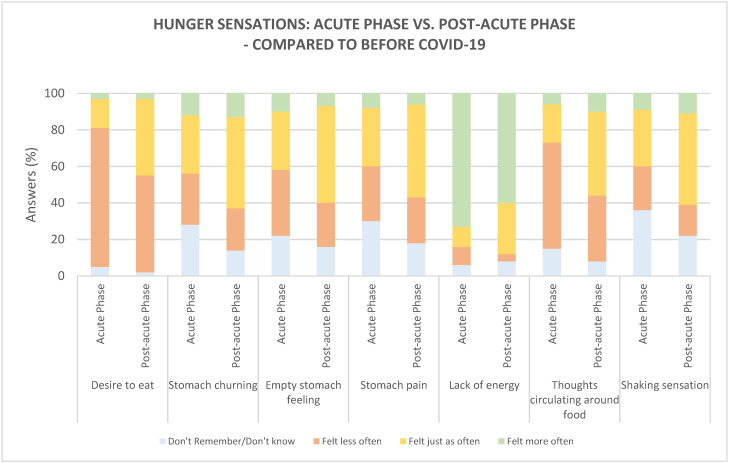
Hunger sensations in the acute phase (*n* = 97) and post-acute phase (*n* = 100), compared to before COVID-19.

The following hunger sensations were explored: desire to eat, stomach churning, empty stomach feeling, stomach pain, lack of energy, thoughts circulating around food, and shaking sensation. Regarding specific hunger sensations, the majority of participants reported ‘less often’ to feel a desire to eat, both during the acute phase (76%) and post-acute phase (53%) of COVID-19, compared to before COVID-19 (Figure 3), and less often to experience that their thoughts circulated around food (in the acute phase only, 58%). The sensations slowly started to return to normal in the post-acute phase. This was found since a significantly higher number of participants (*p* = 0.001, 76% vs. 53%) reported to feel a desire to eat ‘less often’ in the acute phase, and a significantly higher number of participants felt a desire to eat ‘as often as before COVID-19’ in the post-acute phase (*p <* 0.0001, 42% vs. 16%). The same tendency was found for feeling that thoughts circulated around food—a significantly higher number reported to feel the sensation ‘less often’ in the acute phase (*p =* 0.003, 58% vs. 36%) and a significantly higher number reported to feel the sensations ‘as often as before COVID-19’ in the post-acute phase (*p <* 0.001, 46% vs. 21%).

In both phases, the majority of participants (74% in the acute phase and 61% in the post-acute phase) reported ‘more often’ to feel a lack of energy, compared to before COVID-19. A significantly higher number of participants reported feeling a lack of energy ‘as often as before COVID-19’ in the post-acute phase than in the acute phase (*p =* 0.004, 28% vs. 11%), indicating that this sensation slowly returned to normal.

Regarding the physical hunger sensations, stomach churning, empty stomach feeling, stomach pain, and shaking sensation, the majority of participants reported feeling these sensations ‘as often as before COVID-19’ both in the acute phase (ranging from 31% to 32%) and post-acute phase (ranging from 50 to 53%). Comparing the two phases, a significantly higher number of participants reported feeling stomach churning, empty stomach, stomach pain, and shaking sensations ‘as often as before COVID-19’ in the post-acute phase, compared to the acute phase (*p =* 0.015, 50% vs. 32%; *p =* 0.004, 53% vs. 32%; *p =* 0.010, 51% vs. 32%; and *p =* 0.010, 50% vs. 31%, respectively), again indicating that the majority these sensations slowly returned to normal after the acute phase of COVID-19. However, a relatively high percentage of participants (14–36%) showed difficulties remembering these sensations specifically.

**Figure 4:**
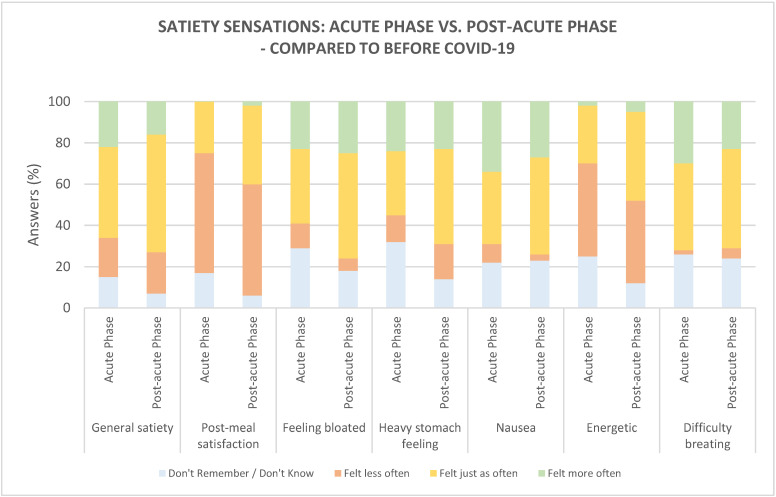
Satiety sensations during the acute phase (*n* = 102) and post-acute phase (*n* = 102), compared to before COVID-19.

The following satiety sensations were explored: general satiety, post-meal satisfaction, feeling bloated, heavy-stomach feeling, nausea, feeling energetic, and difficulty breathing. Regarding the satiety sensations, the majority of participants reported to ‘less often’ feel satisfied after consuming a meal in both the acute phase (58%) and post-acute phase (54%), compared to before COVID-19 (Figure 4). A significantly higher number of participants (*p =* 0.025) showed difficulties in remembering this sensation in the acute phase (17%), compared to the post-acute phase (6%). Additionally, the majority of the participants reported feeling energetic ‘less often’ in the acute phase (45%), and many reported the same during the post-acute phase (40%), compared to before COVID-19. This sensation was found to slowly return to normal since a significantly higher number of participants (*p =* 0.039) in the post-acute phase reported feeling energetic ‘just as often as before COVID-19’ when compared to the acute phase (43% vs. 28%). A significantly higher number of participants (*p =* 0.028) showed difficulty remembering this sensation in the acute phase (25% vs. 12%).

In both phases, participants reported feeling a general satiety ‘as often as before COVID-19’ (44% vs. 57%, respectively). Comparing the two phases, no significant difference was found.

Compared to before COVID-19, participants in both phases reported to ‘more often’ feel the following physical satiety sensations: bloated, heavy stomach, nauseous, and having difficulty in breathing. A significantly higher number of participants reported feeling ‘just as often’ bloated (*p =* 0.047, 51%) and having a heavy stomach (0.042, 46%) in the post-acute phase, compared to the acute phase (36% vs. 31%, respectively), indicating these sensations returning to normal. Further, a significantly higher number of participants (*p =* 0.004) showed difficulty remembering the heavy-stomach sensation in the acute phase (32%), compared to the post-acute phase (14%). Regarding feeling nauseuos and having difficulty breathing, no significant difference was found between the acute and post-acute phases.

As with the hunger sensations, a relatively high percentage of participants (12–32%) showing difficulties in remembering, especially the following sensations, was found: feeling bloated, heavy-stomach feeling, nausea, feeling energetic, and difficulty breathing.

### 3.2. Sensory Perception

#### 3.2.1. Basic Taste Perception and Intake of Food with a Dominant Basic Taste

**Figure 5:**
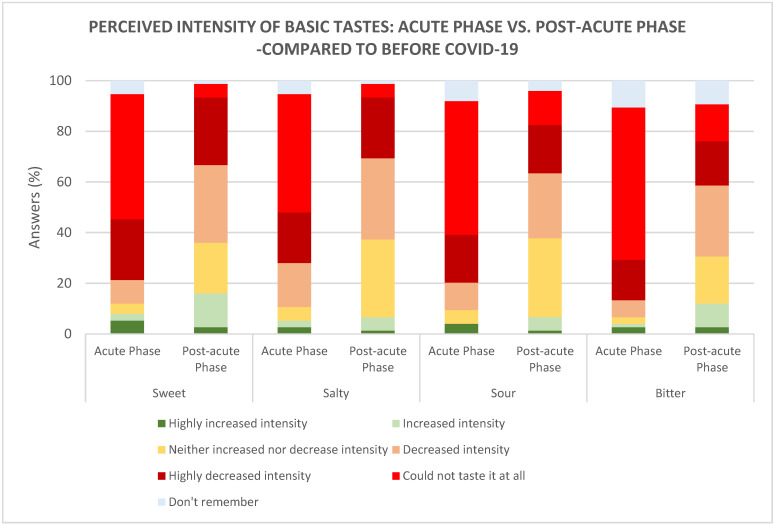
The perceived intensity of the basic tastes during the acute phase (*n* = 75) and post-acute phase (*n* = 75), compared to before COVID-19.

By far, the majority of the participants enrolled in the study reported changes in basic taste perception during the acute phase of COVID-19 as follows: sweet 91%, salty 89%, sour 86%, and bitter 87% (Figure 5). Among the participants who experienced changes in the ability to perceive the basic tastes, around half of the participants reported a total loss in the ability to perceive sweet (49%), salty (46.6%), sour (52.7%), and bitter (60%) tastes during the acute phase, compared to before COVID-19. The remaining participants mainly reported a ’reduced to highly reduced’ ability to perceive the basic tastes, i.e., sweet (33.3%), salty (37.3%), sour (29.7%), and bitter (22.6%). During the post-acute phase, the majority reported a ‘reduced to highly reduced’ basic taste perception, i.e., sweet (57.2%), salty (56%), sour (44.5%), and bitter (45.3%), rather than a total loss, i.e., sweet (5.3%), salty (5.3%), sour (13.5%) and bitter (14.6%). Only a small percentage of the participants reported an ‘increased to highly increased’ sensitivity towards the basic tastes, which was found both in the acute phase, with sweet (8%), salty (5.32%), sour (4%), and bitter (4%), and post-acute phase, with sweet (16%), salty (5%), sour (5%), and bitter (12%). Overall, more of the participants reported a normalised ability to perceive taste in the post-acute phase, compared to the acute phase. This was reflected as a significantly higher ability to perceive sweet (*p <* 0.001), salty (*p <* 0.001), sour (*p <* 0.001), and bitter (*p <* 0.001) in the post-acute phase, compared to the acute phase.

**Figure 6:**
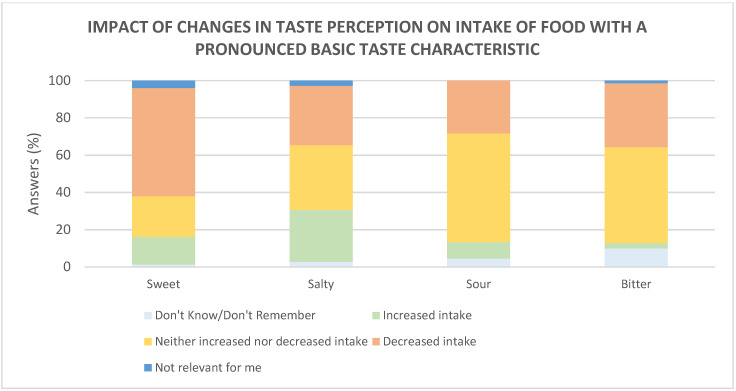
Intake of food with a dominant basic taste after changed perception of basic tastes during the acute phase and post-acute phase, compared to before COVID-19. Sweet (*n* = 74), salty (*n* = 72), sour (*n* = 67), and bitter (*n* = 70).

Among the participants who experienced a change in the perception of basic tastes, the intake of food with a dominant sweet, salty, sour, and bitter taste was evaluated (Figure 6). Regarding intake of food with a dominant sweet, salty, sour, and bitter taste, 58%, 32%, 28% and 34%, respectively, reported to decrease their intake of such foods, whereas 15%, 28%, 9% and 3%, respectively, increased their intake.

#### 3.2.2. Orthonasal and Retronasal Odour Perception 

The majority of the participants (92%) experienced changes in the orthonasal odour perception due to COVID-19. Note that the alteration was not specified for the acute phase and post-acute phase, respectively. Among participants reporting alterations, the majority (64%) reported a complete loss, 34% reported that the odour perception was distorted, 14% reported decreased odour perception, and 7% increased odour perception. More than half of the participants experienced changes in the retronasal odour (67%). Among these, 42% of the participants reported a complete loss of retronasal odour perception, 35% reported that food had a different flavour than usual, and 17% reported a decreased retronasal odour perception. Other (6%) reported odour-specific perception, meaning some odours were perceived retronasally, while others were not.

Participants were asked if they experienced being able to influence the ability to perceive odours orthonasally and retronasally. In both cases, the majority of participants reported ‘not at all’ (orthonasal: 75% and retronasal: 57%), but 14% reported being able to influence orthonasal perception, and 32% retronasal perception (ranging from a ‘small’ to ‘high degree’). To improve the orthonasal perception, participants reported smell training and smelling known scents as the most common strategy. To improve retronasal perception, participants most often added more flavour to meals, e.g., by increasing the number of spices.

**Figure 7:**
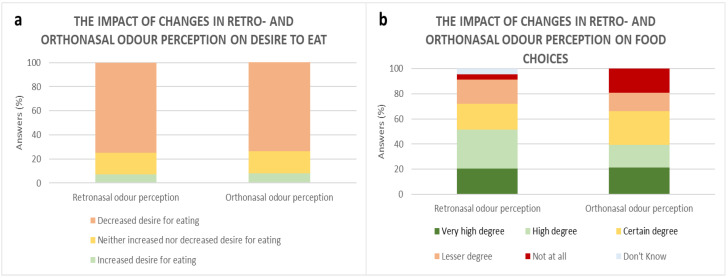
Participants’ report on how changes in retronasal (*n* = 68) and orthonasal (*n* = 92) odour perception, respectively, impacted (**a**) desire to eat and (**b**) food choices.

While participants experienced changes in odour perception, a reduced desire to eat was reported by the majority of participants; among 75% and 80% of the participants experiencing alterations in retronasal and orthonasal odour perception, respectively (Figure 7a). Furthermore, 51% of participants reported that changes in retronasal perception affected their food choices in a ‘high to a very high degree’, 21% to a ‘certain degree’, 19% to a ‘lesser degree’, while around 4% reported that altered flavour perception did ‘not at all’ affect food choices. A total of 39% of participants reported that changes in orthonasal odour perception affected their food choices in a ‘high to a very high degree’, 27% to a ‘certain degree’, 15% to a ‘lesser degree’, while around 19% reported that altered orthonasal perception did ‘not at all’ affect food choices (Figure 7b).

#### 3.2.3. Off-Flavour

A total of 56% of participants reported experiencing off-flavours in the terms of either metallic, rotten, smoked, and/or chemical flavours during food intake. Among these participants, 70%, 27% and 3% reported that these off-flavours ‘reduced’, ‘neither increased nor decreased’, and ‘increased’ their desire to eat, respectively. Furthermore, 43% of participants reported that off-flavours affected their food choices in a ‘high to a very high degree’, 23% to a ‘certain degree’, 21% to a ‘lesser degree’, while around 13% reported that off-flavours did ‘not at all’ affect food choices.

Participants were asked whether they felt they could influence the perceived intensity of off-flavours. A total of 70% reported, ‘not at all’ being able to affect the perceived intensity. Of the 19% who reported that they could affect the perceived intensity of off-flavours, the most often used strategies included rinsing the mouth with water, brushing teeth, chewing mint gum or liquorice, and avoiding intake of foods which provoked the off-flavour.

#### 3.2.4. Chemesthesis

Participants were asked to evaluate whether COVID-19 caused changes in how food felt in the mouth, throat, or gastrointestinal region. Only 26 out of 102 participants reported having experienced changes in perception related to chemesthesis. From reviewing the comments of these participants, it was found that some participants misunderstood the question. Among the participants who understood the question correctly, the following changes were reported: food felt stinging on the tongue and in the throat, difficulty of food to pass through the throat—especially hard/solid foods, and food causing a burning sensation in the mouth and throat. Further, participants reported becoming more sensitive towards the texture of the food when not being able to perceive taste or odours. Especially foods with a soft texture, e.g., oatmeal, mashed potatoes, and boiled vegetables, were reported to cause nausea, and foods with a harder/crunchier texture, e.g., rye bread, apples, and pears, were reported to be more chewable and thereby more comfortable.

Altered chemesthesis during food intake caused a decreased desire for food by the majority of participants experiencing altered chemesthesis (74%). Furthermore, 52% of participants reported that altered chemesthesis affected food choices in a ‘high to a very high degree’, 32% to a ‘certain degree’, 8% to a ‘lesser degree’, while around 8% reported that altered chemesthesis did ‘not at all’ affect food choices. The majority of participants reported experiencing these changes ‘most of the time’ (44%).

### 3.3. Eating Behaviour

#### 3.3.1. Portion Size of Main Meals and Snacks

**Figure 8:**
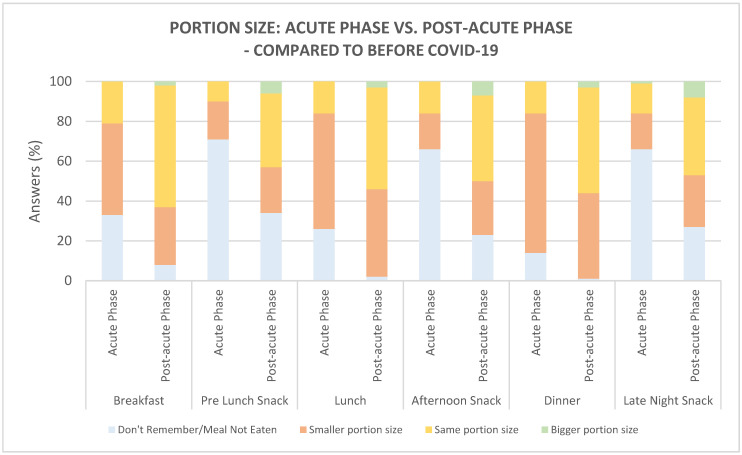
Portion size of meals during the acute phase (*n* = 102) and post-acute phase (*n* = 102), compared to before COVID-19.

The three main meals—breakfast, lunch, and dinner—were eaten by the majority of the participants both during the acute and post-acute phases (Figure 8). Regarding the size of the main meals, the majority reported the meals to be of a ‘smaller size’ in the acute phase compared to before COVID-19 (breakfast: 46%, lunch: 48%, and dinner: 70%). Comparing the two phases, it was found that the breakfast (*p =* 0.02, 46% vs. 29%) and dinner (*p <* 0.001, 70% vs. 43%) were reported to be of a ‘smaller portion size’ by more participants during the acute phase, compared to the post-acute phase. The breakfast, lunch, and dinner (all *p <* 0.001, 61% vs. 21%, 51% vs. 17%, 53% vs. 16%, respectively) were reported to be of ‘the same size as before COVID-19’ by more participants in the post-acute phase, compared to the acute phase. Regarding snack meals eaten pre-lunch, in the afternoon and late night, the majority of participants ‘did not remember/did not eat’ the snacks in the acute phase (pre-lunch: 71%, afternoon: 66%, late night: 66%). During the post-acute phase, more participants reported eating snacks ‘of the same portion size as before COVID-19’ than in the acute phase (all snack meals *p <* 0.001).

#### 3.3.2. Type of Diet

**Figure 9:**
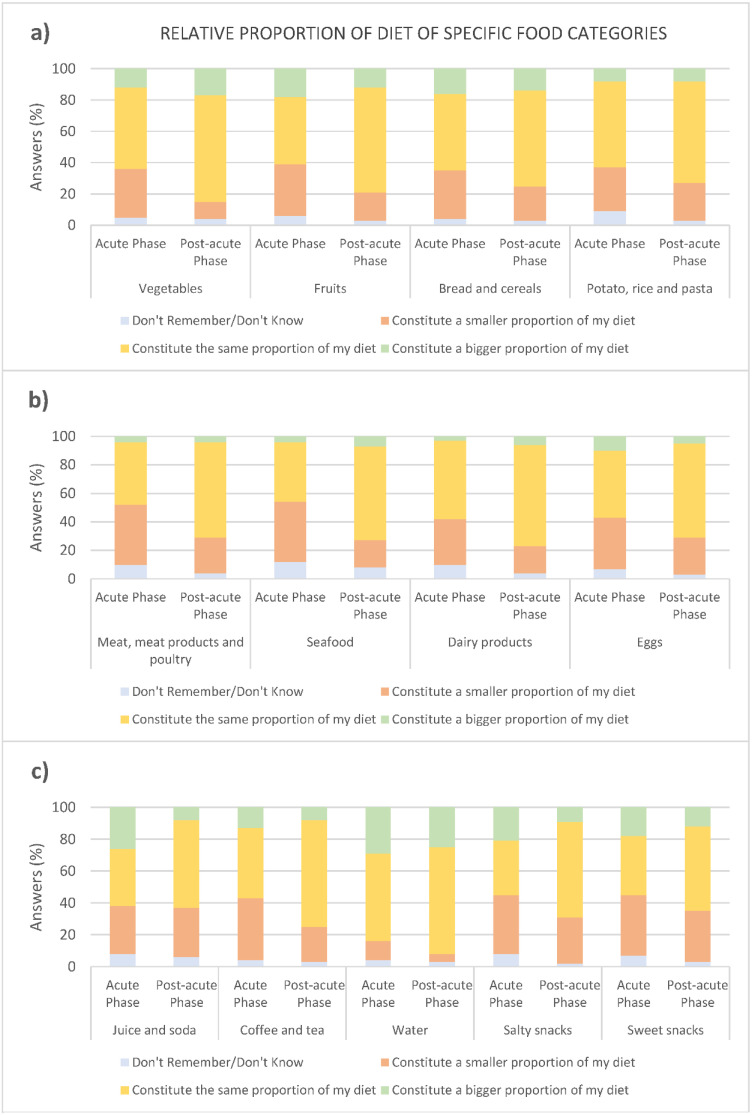
Type of diet during the acute phase (*n* = 102) and post-acute phase (*n* = 102), compared to before COVID-19. (**a**) Vegetables, fruits, and starchy foods; (**b**) animal products; and (**c**) drinks and snacks.

Vegetables, fruits, and starchy foods: During the acute phase, between 28% and 33% of participants reported vegetables, fruits, and starchy foods (bread and cereals, rice, potato, and pasta) to constitute ‘a smaller proportion of the diet’, compared to before COVID-19, and 43–55% reported the foods to constitute ‘the same proportion of the diet’, compared to before COVID-19 (Figure 9a). Comparing the two phases, a significantly higher number of participants reported vegetables (*p =* 0.001) and fruits (*p =* 0.023) to constitute ‘a smaller proportion of their diet’ during the acute phase (vegetables: 31% and fruit: 33%) than during the post-acute phase (vegetables: 11% and fruit: 18%). A significantly higher number of participants reported vegetables (*p =* 0.033) and fruits (*p =* 0.001) to constitute ‘the same proportion of their diet’ during the post-acute phase (vegetables: 68% and fruit: 67%), compared to the acute phase (vegetables: 52% and fruit: 43%). These results indicate that the intake of vegetables and fruit starts to return to normal during the post-acute phase. No significant difference was found for starchy foods between the two phases.

Meat, seafood, dairy products, and eggs: The majority of participants reported meat, meat products and poultry (42%), seafood (42%), dairy products (32%), and eggs (36%) to constitute a ‘smaller proportion of the diet’ during the acute phase, compared to before COVID-19 (Figure 9b). A significantly higher number of participants reported a smaller intake of meat, meat products and poultry (*p =* 0.017), and seafood (*p =* 0.001) during the acute phase, compared to the post-acute phase (25%, 19%, 19%, and 26%, respectively). For all four food categories, a significantly larger number of participants reported that the food constituted the ‘same proportion of diet’ during the post-acute phase, compared to the acute phase: meat, meat products and poultry (*p =* 0.002, 67% vs. 44%), seafood (*p =* 0.001, 66% vs. 42%), dairy (*p =* 0.030, 71% vs. 55%) and eggs (*p =* 0.011, 66% vs. 47%).

Beverages: A total of 27%, 36%, and 30%, respectively, reported juice to constitute ‘a bigger’, ‘the same’, and ‘a smaller’ proportion of the diet in the acute phase of COVID-19, compared to before COVID-19 (Figure 9c). More reported juice intake to be ‘larger’ during the acute phase, compared to the post-acute phase (*p =* 0.002, 27% vs. 9%), and more reported the intake to be ‘the same’ in the post-acute phase, compared to the acute phase (*p =* 0.011, 55% vs. 36%). An approximate even number of participants reported coffee/tea to make up ‘a smaller’ and ‘the same’ proportion of the diet (39% and 44%, respectively) during the acute phase. The intake started to return to normal during the post-acute phase. This was observed by more participants who reported the intake to constitute ‘a smaller proportion of the diet’ in the acute phase, compared to the post-acute phase (*p =* 0.014, 39% vs. 22%), and more participants reported the intake to constitute ‘the same proportion of diet’ during the post-acute phase (*p =* 0.001, 67% vs. 44%). The intake of water was, by the majority, ‘the same’ in the acute and post-acute phases (55% and 67%, respectively), compared to before COVID-19, and no significant differences were found between the two phases. A total of 30% and 26% reported that their intake of water constituted ‘a bigger proportion of the diet’ in the acute phase and post-acute phase, respectively.

Salty and sweet snacks: Intake of salty snacks was reported to constitute ‘a bigger’, ‘the same’, and ‘a smaller’ proportion of the diet, compared to before COVID-19 by 23%, 34%, and 37% of participants, respectively (Figure 9c). The number of participants who reported the intake to be ‘bigger’ was larger during the acute than the post-acute phase (*p =* 0.038, 23% vs. 11%), whereas the number of participants who reported their intake to be ‘the same as before COVID-19’ was larger during the post-acute phase (*p <* 0.001, 60% vs. 34%). The relative proportion of sweet food was reported to be ‘bigger’, ‘the same’, and ‘smaller’, compared to before COVID-19 by 20%, 37%, and 38% of participants, respectively. The number of participants reporting the intake to constitute ‘the same’ proportion of the diet was significantly higher in the post-acute phase, compared to the acute phase (*p =* 0.024, 53% vs. 37%), during which around half (53%) reported sweet food to constitute the same proportion of the diet as before COVID-19.

#### 3.3.3. Texture, Temperature, and Preparation Method

**Figure 10:**
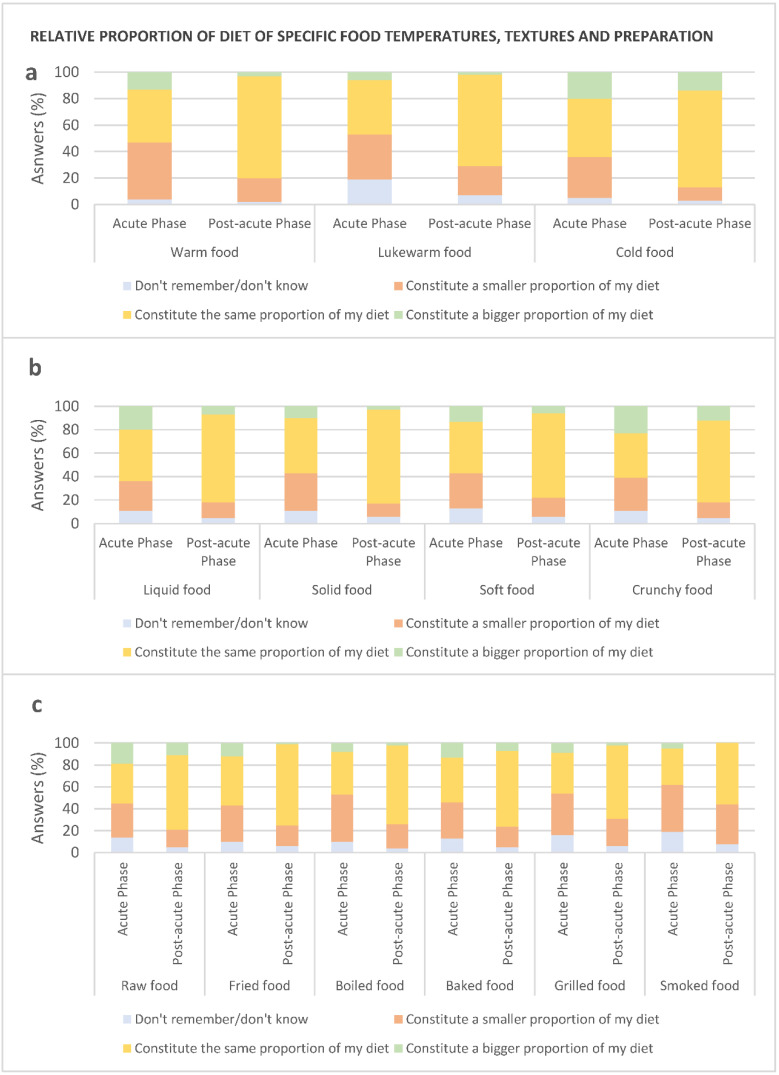
Type of food during the acute phase (*n* = 102) and post-acute phase (*n* = 102), compared to before COVID-19. (**a**) Temperature of food, (**b**) texture of food, and (**c**) cooking.

Temperature: Regarding the temperature of food, a relatively high percentage of participants reported that warm- (43%), lukewarm- (34%), and cold food (31%) constituted a ‘smaller proportion’ of the diet during the acute phase, compared to before COVID-19 (Figure 10a). All three temperatures were reported to constitute the ‘same proportion’ of the diet by the majority of the participants during the post-acute phase (77%, 69%, and 73%, respectively). Comparing the two phases, a significantly higher number of participants reported warm- (*p <* 0.001, 43% vs. 18%) and cold food (*p <* 0.001, 31% vs. 10%) to constitute a ‘smaller proportion’ during the acute phase, and warm- (*p <* 0.001, 77% vs. 40%), lukewarm- (*p <* 0.001, 69% vs. 41%), and cold food (*p <* 0.001, 73% vs. 44%) to constitute ‘the same proportion’ in the post-acute phase, compared to the acute phase. A significantly higher number of participants (*p =* 0.020, 19% vs. 7%) showed difficulty in remembering their consumption of lukewarm food during the acute phase, compared to the post-acute phase. Comparing the three temperatures, in both phases, more people reported cold food to ‘constitute a bigger proportion of my diet’ (acute: 16%, post-acute: 22%) than warm- (acute: 15%, post-acute: 5%) and lukewarm food (acute: 8%, post-acute: 4%). However, also intake of warm food was found to constitute ‘a larger part of the diet’ in the acute phase for some participants (15%). During the post-acute phase, this number significantly dropped to 5% (*p =* 0.032).

Texture: The majority of the participants reported the textures; liquid (44%), solid (47%), soft (44%), and crunchy (38%) food to constitute ‘the same proportion’ of the diet during the acute phase, compared to before COVID-19 (Figure 10b). The intake of all types of textures, was slowly returning back to normal in the post-acute phase, during which a significantly higher number of participants reported liquid (*p <* 0.001, 75% vs. 44%), solid (*p <* 0.001, 80% vs. 47%), soft (*p <* 0.001, 72% vs. 44%), and crunchy (*p <* 0.001, 70% vs. 38%) to constitute ‘the same proportion’ of the diet, compared to before COVID-19. Comparing and observing the texture for which most participants showed an ‘increase´, it was found that intake of crunchy and liquid food was increased by the largest number of participants (24% and 21%, respectively) in the acute phase.

Preparation method: Regarding the preparation method chosen, a relatively high percentage of participants reported boiled- (43%), smoked- (43%), and grilled food (38%) to constitute ‘a smaller proportion’ of the diet during the acute phase, compared to before COVID-19 (Figure 10c)**.** For, the remaining preparation methods—raw, fried, and baked food—31–33% reported the preparation methods to constitute ‘a smaller proportion’ of the diet. A significant higher number of participants reported the preparation method (beside smoked) to constitute a ‘smaller proportion’ of the diet during the acute phase, compared to the post-acute phase: raw- (*p =* 0.013, 31% vs. 16%), fried- (*p =* 0.024, 33% vs. 19%), boiled- (*p =* 0.002, 43% vs. 22%), baked- (*p =* 0.036, 33% vs. 19%), and grilled food (*p =* 0.048, 38% vs. 25%). Further, smoked- (*p =* 0.028, 5% vs. 0%) and fried food (*p =* 0.016, 12% vs. 3%) was found to ‘constitute a bigger proportion’ of the diet during the acute phase, compared to the post-acute phase. During the post-acute phase, the majority of participants reported all preparation methods to constitute ‘the same proportion’ as before COVID-19, and from the acute phase to the post-acute phase, the number of participants reporting the preparation methods to constitute ‘the same proportion’ as before COVID-19 increased (raw (*p <* 0.0001, 36% vs. 68%), fried (*p <* 0.001, 45% vs. 74%), boiled (*p <* 0.001, 39% vs. 72%), *baked* (*p <* 0.001, 41% vs. 69%), grilled (*p <* 0.001, 37% vs. 67%), and smoked (*p <* 0.001, 33% vs. 58%)). Comparing the preparation methods and observing the preparation method for which most participants showed an ´increase´, it was found that intake of raw- and baked food was increased by the largest number of participants (20% and 14%, respectively) in the acute phase.

### 3.4. Handling of Changes in Appetite, Sensory Perception, and Food Behaviour

**Figure 11:**
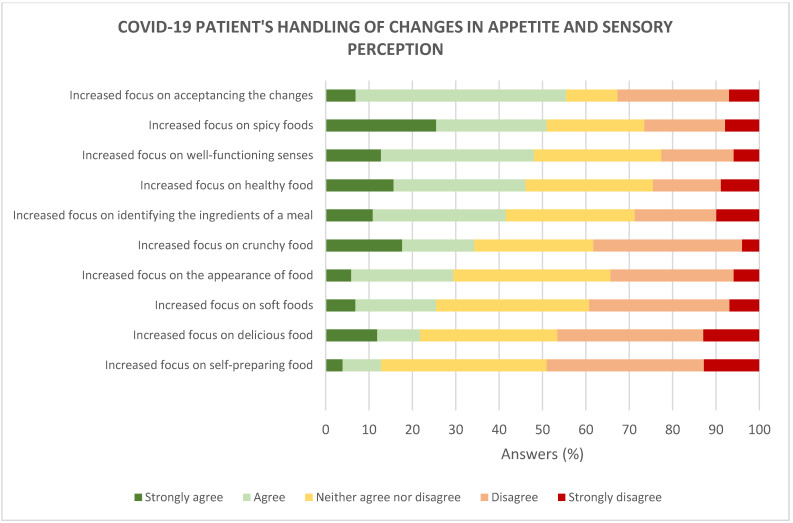
Participants’ agreements on strategies for boosting appetite and sensory perception (*n* = 102).

Participants were asked to report their agreement on trying different strategies for handling changes in appetite, sensory perception, and eating behaviour (Figure 11). The list of strategies was developed based on a qualitative study with patients COVID-19, conducted prior to this online survey [20].

Overall, accepting changes in appetite and sensory perception was the strategy that was used mostly by the participants (56%) during the whole period of COVID-19 disease and recovery. While experiencing changes in appetite and/or sensory perception, the majority of participants agreed that it was important to be able to identify every ingredient in a meal (42%) and to increase their focus on senses that were well functioning (48%). From a food-behavioural perspective, the majority agreed to increase their focus on eating spicy foods (50%), and on eating healthy food (46%), whereas the majority disagreed to increase their focus on delicious foods (47%) and self-preparing foods (49%). When asked about the increased focus on crunchy food and the appearance of food, participants were equally split into three groups indicating to ‘agree’, ‘neither agree nor disagree’ and ‘disagree’ (compared to before COVID-19).

## 4. Discussion

### 4.1. Altered Appetite

Loss of appetite is a well-documented consequence of several illnesses including various influenzas and colds [26], and recently, COVID-19 [3,20,27]. The present study presents confirmatory evidence for the effects of COVID-19 on appetite during the acute phase of the disease. Further, the study contributed to new knowledge by addressing the specific hunger and satiety sensations and by showing how the specific appetite-altering effects of COVID-19 continue into the post-acute phase of the COVID-19 disease. Among the list of symptoms presented in this online survey, lack of hunger sensations was reported to be among the main cause of reduced appetite in both the acute phase and post-acute phase of COVID-19, during which participants reported experiencing less often a desire to eat and to have thoughts circulating around food and more often feeling a lack of energy. Similar findings were found in a qualitative study on appetite among patients showing long-term effects of COVID-19 [20]. Through in-depth interviews, the COVID-19 patients expressed a lack of hunger sensations, compared to before COVID-19, and a faster fullness during the consumption of a meal resulting in reduced food intake [20]. Supporting these findings, the present study likewise found that participants more often experienced overall satiety, characterised by more often feeling bloated, heavy stomach feeling, nauseous, and difficulty in breathing, explaining the reduction in appetite.

Besides the lack of hunger sensations, participants in the current study reported that changes in chemosensory perception, i.e., alterations in taste and smell perception were among the main causes of alterations in appetite. Perception of food’s sensory properties is highly linked to hunger and satiety sensations experienced during a meal and therefore play an important role in food intake control [28,29]. During the early stage of a meal, the sensory properties of food generate a positive feedback mechanism, i.e., liking of sensory properties enhance hunger and drive continued intake [30]. During the later stages of a meal, hunger sensations decline, and satiation takes over. Experiencing impairments in the ability to perceive taste and aroma properties can hinder the early stage positive feedback, resulting in a faster decrease in hunger and/or onset of satiation, experienced as a general overall satiation and lack of hunger, explaining the altered appetite by the participants in the present study. Similar results were found in a qualitative study by Høier and colleagues [20], in which COVID-19 patients expressed a lack of interest in food due to food being tasteless and not being able to smell the food while cooking to stimulate appetite. Supporting this view, a study by Merkonidis et al. [31] found that most individuals suffering from chemosensory disorders showed altered eating behaviour, reduced food intake, and/or changed food preferences. The reduced food intake was caused by a lack of sensory cues such as the smell, sight, and taste of food, which normally would motivate eating [31,32,33]. Further, it was found that the most common complaint among individuals with chemosensory disorders was the loss of pleasure from eating [31]. In line with these results, participants in the present study reported a diminished pleasure from eating and indicated alterations in the taste of food (to be understood as flavour) to be among the main causes.

Although feeling generally satiated, the majority of the participants, at the same time, reported feeling satisfied less often after consumption of a meal. These results can be explained by the phenomenon of sensory satisfaction [15]. Sensory satisfaction describes how the perception of sensory properties during food consumption, fulfils desires in a meal experience and can lead to a feeling of postmeal satisfaction [15,16,34,35]. Losing the ability to perceive the sensory properties of food changes the sensory experience of a meal during consumption, which can lead to the meal being perceived as less satisfying. This phenomenon is related to the hedonic aspects of food intake, which indicates that satiety is driven by both homeostatic factors and the fulfilment of hedonic desires, and the perception of sensory properties plays a causal role in satisfying these desires [35]. In the qualitative study by Høier, Chaaban, and Andersen [20], participants expressed the lack of satisfaction from eating as feeling ‘unpleased senses’, and for a smaller group, chemosensory dysfunction led to continued eating in order to find foods that could satisfy their sensory desires. Merkonidis et al. [31] likewise approached the association between sensory perception and intake. They found that a smaller percentage of individuals suffering from chemosensory disorders increased their food intake in the search for flavour in their meals. It can therefore be hypothesised that a lack of feeling sensory satisfied drove the increased desire for food, reported by a minority of participants in the present study.

### 4.2. Sensory Perception

Since the outbreak of the COVID-19 pandemic, several studies aimed to measure the prevalence and severity of chemosensory alterations among COVID-19 patients, either based on self-reporting [3,5,7,8,36] or by objective chemosensory testing [4,9,11,12,37,38]. Suffering from olfactory and gustatory dysfunction is now considered one of the most prevalent symptoms of COVID-19, ranging between 5 and 89% of the patients’ complaints [36]. Data from a systematic review on COVID-19 patients showed a high global prevalence of smell (48%) and taste dysfunction (41%) and a combination of both (35%) [39]. The present study contributed to this research by characterising the chemosensory dysfunctions. It should be noted that the percentage of participants reporting chemosensory dysfunction generally is higher in the present study, compared to previous literature, due to the inclusion criteria of this study. Alterations were found for all basic taste attributes (sweet, salty, sour, and bitter) and included a total loss of basic taste perception (ageusia) and altered intensity in taste perception, in the order ageusia (total loss) > hypogeusia (decreased perception) > hypergeusia (increased perception). A study by Parma et al. likewise found impairments in two or more taste qualities among participants suffering from COVID-19 [8].

Alterations in smell perception were characterised by a total loss of smell perception (anosmia) and odours becoming altered and unpleasant (parosmia), in the order of more experiencing anosmia (total loss) > parosmia (distorted) > hyposmia (decreased perception) > hyperosmia (increased perception). The qualitative changes in sense of smell confirm findings from Parma et al., who likewise reported anosmia, parosmia, and hyposmia as well as phantosmia. Further, in the present study, a total loss of retronasal odour perception and distorted flavour perception was found, which can be regarded as a combined dysfunctional flavor and taste perception.

Previous studies have stated that chemosensory disorders begin during the early stages of COVID-19 disease [36,37,38], which was confirmed by the present study. Although the loss of smell and taste function are common in acute cold, according to a study by Huart et al. [38], the effects on chemosensory dysfunction are more severe when caused by COVID-19, compared to acute colds, since both smell identification scores and taste function were found to be significantly lower in COVID-19 patients, compared to acute cold patients [38].

When reviewing existing literature, it is indicated that recovery of gustatory and olfactory disorders most often occurs within few weeks after infection, while in some cases, the recovery process will be longer [36,37]. Only participants in the post-acute phase of COVID-19 disease were included in the present study, and therefore, the time period for recovery was not the focus. However, comparing participants’ self-reported perception of the basic tastes during the acute and post-acute phases, a slow recovery of taste function was found. Interestingly, more participants reported still suffering from altered ability to perceive sweet and bitter tastes, compared to salt and sour tastes, during the post-acute phase, indicating a slower recovery of sweet and bitter taste perception. Similar findings have been reported by Huart et al. [38], who investigated taste (global, sweet, sour, bitter, salty) and odour perception amongst patients recovering from COVID-19 and acute cold. Focusing on taste perception, they found worse global, sweet, and bitter perception, amongst COVID-19 recovering patients, whereas there were no differences in perception of sour and salt perception.

### 4.3. Eating Behaviour

COVID-19 disease is believed to put patients at risk of malnourishment since several illness-related factors including nausea, diarrhea, loss of appetite, loss of taste and smell, and stress over time can cause a reduction in food intake and the nutritional value of the diet [39,40]. As discussed previously, the majority of the participants reported a reduction in appetite during the acute phase, compared to before COVID-19. This reduction in appetite was observed at all times of the day—in the morning, in the forenoon, at lunchtime, in the afternoon, in the eve, and at late night (see Section 3.1.2). Participants further reported reductions in food intake due to COVID-19. At all times of day, main and snack meals (i.e., breakfast, pre-lunch snack, lunch, afternoon snack, dinner, and late-night snack) were reported to be of smaller portion size, or not eaten at all/did not remember. A preference towards the three main meals, i.e., breakfast, lunch, and dinner, compared to the snack meals was found, since the majority of the participants reported eating these meals, although a smaller portion size. During the post-acute phase, the desire for food was, in general, also reported to be highest around the three main meals, compared to the snack meals. This was likewise reflected in the portion size since the majority of the participants reported the main meals to be of ‘the same size’ as before COVID-19, although a high proportion still reported the main meals to be of a smaller size.

These results provide evidence of COVID-19 affecting the quantitative aspect of food intake and also confirm that the desire for food plays an important role in the motivation for food intake. To the authors’ knowledge, this study is the first to investigate the effect of COVID-19 on food intake. Although many participants were still suffering from reduced appetite during the post-acute phase, the majority improved their food intake, which aligns with the participants’ self-reports of the normalisation of their appetite. It is noticeable that even in the acute phase, participants showed a preference for the three main meals, indicating that the participants were aware of the importance of food in the process of recovery. This result brings opportunities for health professionals, such as dieticians, to focus on the main meals when pushing health-related initiatives and be aware of reduced appetite outside these meals.

In the present study, participants were asked to report on qualitative changes in their diet by evaluating if food categories during the acute and post-acute phases constituted an altered proportion of the diet. As discussed in the previous section, participants, in general, showed a reduced food intake at all times of day during the acute phase of COVID-19. This finding was reflected in an overall reduction in intake of various food categories. Most of the participants reported a normalised intake of all food categories during the post-acute phase.

During the acute phase of COVID-19, the intake of meat, seafood, eggs, coffee/tea, salty and sweet snacks was reduced. Regarding the food category ‘meat, seafood, and egg’, participants reported disliking these foods due to not being able to perceive the flavour, resulting in a greater awareness of the texture/consistency of this food category, which was not perceived as pleasant. Eating is a multi-sensory experience based on the perception of sensory properties of food, i.e., taste, smell, appearance, and texture. Taste (understood as flavour) has previously been found to be the main driver of hedonic eating experience [28] and more important for the hedonic aspect of food, compared to texture, smell, and appearance [41,42,43]. The results from the present study highlight the pronounced importance of flavour for food acceptance, in this case, meat, seafood, and egg acceptance specifically, since when taste and retronasal odour perception suffered, participants shifted focus towards the texture as the driver of food acceptance. The shift in focus towards other sensory properties of food when not being able to perceive taste and smell properties has previously been shown among individuals diagnosed with chemosensory disorders [31] and COVID-19 recovering patients [20]. The studies reported that participants obtained food-related pleasure by focusing on chemesthesis via the well-functioning sense of touch. In practice, this was achieved via the perception of the food’s texture, especially by adding crunchy elements [20], and trigeminal stimulation by adding spices to food [20,44]. The increased preference for crunchy food was further reflected in the results concerning preparation methods, since an increased intake of raw food was found in the present study. Raw food is generally associated with a crunchier texture than heat-treated food. Soft textures and boiled food which generally is associated with a softer texture were reported to be less preferred during the acute phase. The present study thereby provides confirmatory evidence for focusing on chemesthesis (especially via crunchy and spicy food) to cope with taste and odour alterations, maintain food-related pleasure, and highlight actionable opportunities for individuals and health professionals interested in boosting appetite and eating enjoyment.

In the present study, sweet and salty snacks were found to constitute an altered, most often smaller, part of the diet when suffering from COVID-19, and pointed at altered basic taste perception to be (to some degree) the direct cause of altered intake of food with a dominant sweet, salty, sour, and bitter taste. Individuals experiencing chemosensory disorders commonly report changed eating behaviour [31,45], but mixed results have been reported about the association between intake quality and altered chemosensory function. One study showed a change in diet towards a more Western-style diet—high in fast food, sweet, salty, and/or fats—when suffering from olfactory dysfunction [44]. Other studies have shown a reduced intake of sweet and fatty food [46,47] and in one study also salty food [48] with a preference towards fruits and vegetables, indicating a shift towards healthier food choices. The mixed results per se, regarding changes in qualitative aspects of the diet when suffering from chemosensory dysfunction, have likewise been pointed out previously. The study by Høier, Chabaan, and Andersen [20] suggested that individuals suffering from chemosensory dysfunction can be divided into two groups—a smaller group reporting to increase intake of unhealthy food since these foods reminded them of past pleasurable experiences upon consumption of these foods, and a larger second group, reporting to focus on healthy eating since this was associated with higher mental well-being when providing the body with beneficial nutrients. The indication of two groups, showing opposite eating behavioural changes, was supported by the present study; 20% and 23% reported to increase their intake of sweet and salty snacks, respectively, and 38% and 37% reported to decrease their intake of sweet and salty snacks, respectively, as a consequence of COVID-19.

Finally, the study provides confirmatory evidence of off-flavours occurring during COVID-19 disease. Approximately half of the participants in the present study reported experiencing off-flavours, supporting previous findings by Høier et al. [20]. The off-flavours were characterised as metallic, rotten, smoked, and/or chemical flavours during food intake and were reported to lower participants’ desire to eat and to some extend also to impact food choices. The characteristics of the off-flavours can explain why a relatively high percentage of participants reported smoked- (43%) and grilled food (38%) to constitute ‘a smaller proportion’ of the diet.

### 4.4. Limitations

The present study constitutes an online survey based on subjective reports. This approach offers some possibilities along with some limitations. A limitation when stating findings from the acute phase of COVID-19 is that the outcome is strongly dependent on participants’ memory. Since all participants were in the post-acute phase of COVID-19 when conducting the study, retrospection was necessary but may not always be accurate. Asking multiple questions to the same response variable would allow a check of reliability. In the present study, one question per response variable was chosen under consideration of the length of the questionnaire and to avoid fatigue. The use of open-reply fields after each topic allowed the researchers to check if participants had understood the questions. Further, although the patient perspective is indeed relevant since sensations, in many cases, drive human behaviour, the findings could preferably be supported by objective means. Sensory perception could be validated, e.g., by the use of threshold tests, and eating behaviour could be validated by the use of dietary records.

Since the study aimed to provide a detailed characterisation of the appetite, sensory perceptional, and eating behavioural effects of COVID-19 disease, only participants experiencing these alterations were included in the study. Thereby, the present study does not address the prevalence of these symptoms among COVID-19 patients. Although the current study did not study nor suggest gender differences, it should be noted that the study population consisted of 88% females. A study with a bigger sample size including more males could preferably be conducted to check generalisability of the results.

### 4.5. Application of Findings and Suggestions for Future Research

The study brings results that can be applied by health professionals to secure eating enjoyment and thereby nutrition when suffering from altered taste and odour perception. Specifically, this article can serve as a basic information document for dietetic guidance, in which health professionals can seek information about normal occurring appetite, sensory perceptional, and eating behavioural changes due to COVID-19, along with inter-individual differences. Further, the article points to ways of applying the findings in dietary guidance. For example, a key strategy to secure eating enjoyment is to work with the drivers of pleasure. Consciously shifting focus from flavour as primary driver of consummatory pleasure towards the more well-functioning sense of touch proves to be a good strategy. In practical terms, this can be achieved via an emphasis on food textures, e.g., variation in crunchiness, by the use of raw vegetables, and/or variation in trigeminal stimulation by the use of irritants such as hot spices. However, the mental barriers and possibilities preventing/supporting these mental shifts need further study. Additionally, strategic use of healthy vs. unhealthy food to support intake and pleasure needs further exploration. Therefore, future studies should focus on how to apply these findings among COVID-19 patients, and if/how they can be broadened to other patients groups suffering from chemosensory disorders, in order to maintain appetite and secure nutrition.

## 5. Conclusions

This study aimed to investigate, the acute and long-term effects of COVID-19 disease on details of appetite, sensory perception, and eating behaviour. Key results revealed that although the majority reported eating behaviour and sensory perceptions to return to baseline as experienced before COVID-19, many still in the post-acute phase of the disease reported altered appetite.

The desire for food was reported severely impaired by the majority of participants during both the acute phase and post-acute phase, compared to before COVID-19. The low desire for food was, for many, associated with altered appetite and satiety sensations, and when engaging in consumption, the majority of participants were less often left with a satisfied feeling.

Basic taste- and orthonasal odour perception, were by the majority of participants, reported to be reduced due to COVID-19, and retronasal odour perception was reported to be reduced among approximately half of the participants. The changes were often characterised by a complete loss or disordered perception, along with the experience of off-flavours. Sensory alterations were reported a reduction in the desire to eat and affect food choices, yet some participants found themselves capable of improving ortho- and retronasal odour perception.

Eating focused on the three daily main meals (not snacks), both in the acute phase and post-acute phase, and meals were for many of smaller portion size. In general, during the acute phase, the majority reported alterations in the food types included in the diet, and around half reported alterations in the choice of preparation method, the texture of meals, and choice of warm–cold meals. The relative proportion of the different types of food and beverages was comparable to before COVID-19 for the majority of participants in the post-acute phase. The same tendency was found regarding the temperature of meals, preparation method, and different textures.

To cope with the changes in appetite and sensory perception, participants focused on changing their focus during eating—from a focus on taste and smell to the more well-functioning senses, e.g., touch. For many, this resulted in an increased focus on the intake of spicy, healthy, and crunchy foods.

Altogether, the findings from this study illustrate the complexity by which COVID-19 affects human appetite and sensory perception and points toward strategies to cope with these changes. Future studies could by advantage focus on validation of the results via objective measurements and application of the findings in dietary interventions for people suffering from sensory- and appetite-related impairments including COVID-19 patients.

## Figures and Tables

**Table 1:** Participant characteristics.

Characteristics	
Total number (*n*)	102
Males/females	14/88
Age (years)	41 ± 12.9 (19–69) *
BMI (kg/m^2^)	25.7 ± 5 (17.8–42.9) *
Educational level (min–max) **	4.7 ± 1.3 (2–6) *
Inhabitants/household (number of persons)	3 ± 1.4 (1–8) *

* Mean ± standard deviation (range). ** Educational level: (1) lower secondary, (2) higher secondary, (3) higher secondary with trainee, (4) short-length higher education, (5) medium-length higher education, and (6) long higher education.

**Table 2:** Response variables used in the online survey.

Response Variable	During the Acute Phase	During the Post-Acute Phase
Desire for food	‘During the acute phase, how large was your desire for food compared to before COVID-19?’	‘While you are in the post-acute phase, how large has your desire for food been recently compared to before COVID-19’
Hunger	‘Indicate how COVID-19 affected following hunger sensations, x, during the acute phase (compared to before COVID-19)’ x = ‘desire to eat’,’ stomach churning’, ‘empty stomach feeling’, ‘stomach pain’, ‘lack of energy’, ‘thoughts circulating around food’ and ‘shaking sensation’	‘Now that you are in the post-acute phase, how will you assess following hunger sensations, x, compared to before COVID-19?’ x = ‘desire to eat’, ‘stomach churning’, ‘empty stomach feeling’, ‘stomach pain’, ‘lack of energy’, ‘thoughts circulating around food’ and ‘shaking sensation’
Satiety	‘Indicate how COVID-19 affected following satiety sensations, x, during the acute phase (compared to before COVID-19)’ x = ‘general satiety’, ‘post-meal satisfaction’, ‘feeling bloated’, ‘heavy stomach feeling’, ‘nausea’, ‘energetic’ and ‘difficulty breathing’	‘Now that you are in the post-acute phase, how will you assess following satiety sensations, x, compared to before COVID-19?’ x = ‘general satiety’, ‘post-meal satisfaction’, ‘feeling bloated’, ‘heavy stomach feeling’, ‘nausea’, ‘energetic’ and ‘difficulty breathing’
Taste perception	‘During the acute phase, how did you experience the intensity of the x taste?’ x = ‘sweet’, ‘salty’, ‘sour’ and ‘bitter’	‘How are you experiencing the intensity of the x taste lately?’ x = ‘sweet’, ‘salty’, ‘sour’ and ‘bitter’
Retronasal odour perception	‘Did COVID-19 change your ability to perceive flavours?’ ‘How did the changes ability of perceiving flavour affect your desire for eating?’	
Off-flavour perception	‘Did COVID-19 cause any off-flavours in your mouth?’ ‘How did these off-flavours affect your desire for eating?’	
Orthonasal odour perception	‘Did COVID-19 change your ability to perceive odours?’ ‘How did the changes in the ability of perceiving odours affect your desire for eating?’	
Chemesthetic perception *	‘Did COVID-19 cause any changes in the sense of touch during food intake?’ ‘How did these feelings affect your desire for eating?’	
Quantitative food intake	The participants were asked to indicate the portion size of their daily meals (x) during the acute phase compared to before COVID-19. x = ‘breakfast’, ‘pre-lunch snack’, ‘lunch’, ‘afternoon snack’, ‘dinner’, ‘late night snack’	The participants were asked to indicate the portion size of their current daily meals (x) compared to before COVID-19. x = ‘breakfast’, ‘pre-lunch snack’, ‘lunch’, ‘afternoon snack’, ‘dinner’, ‘late night snack’
Qualitative food intake	The participants were asked to indicate to what extent a certain food and type of food, x, were part of their diet during the acute phase compared to before COVID-19. x = ‘vegetables’, ‘fruits’, ‘bread and cereal’, ‘pasta, rice and potato’, ‘meat, meat products and poultry’, ‘seafood’, ‘dairy products’, ‘eggs’, ‘juice and soda’, ‘coffee and tea’, ‘water’, ‘salty snacks’ and ‘sweet snacks’	The participants were asked to indicate to what extent a certain food and type of food, x, are part of their diet currently compared to before COVID-19. x = ‘vegetables’, ‘fruits’, ‘bread and cereal’, ‘pasta, rice and potato’, ‘meat, meat products and poultry’, ‘seafood’, ‘dairy products’, ‘eggs’, ‘juice and soda’, ‘coffee and tea’, ‘water’, ‘salty snacks’ and ‘sweet snacks’

* Defined as food-caused chemesthetic sensations in the mouth and/or gastrointestinal region.

## Data Availability

The data presented in this study is available on request from the corresponding author.
